# Characterizing Cycling Smoothness and Rhythm in Children With and Without Cerebral Palsy

**DOI:** 10.3389/fresc.2021.690046

**Published:** 2021-09-07

**Authors:** Ashwini Sansare, Ahad Behboodi, Therese E. Johnston, Barry Bodt, Samuel C. K. Lee

**Affiliations:** ^1^Department of Physical Therapy, University of Delaware, Newark, DE, United States; ^2^Rehabilitation Medicine Department, Clinical Center, National Institutes of Health, Bethesda, MD, United States; ^3^Department of Physical Therapy, Jefferson College of Rehabilitation Sciences, Thomas Jefferson University, Philadelphia, PA, United States; ^4^Biostatistics Core Facility, University of Delaware, Newark, DE, United States

**Keywords:** recumbent cycling, rehabilitation, physical activity, motor control, fitness

## Abstract

Stationary cycling is a practical exercise modality in children with cerebral palsy (CP) that lack the strength for upright exercises. However, there is a lack of robust, sensitive metrics that can quantitatively assess the motor control during cycling. The purpose of this brief report was to characterize the differences in motor control of cycling in children with CP and with typical development by developing novel metrics to quantify cycling smoothness and rhythm. Thirty one children with spastic diplegic CP and 10 children with typical development cycled on a stationary cycle. Cycling smoothness was measured by cross-correlating the crank angle with an ideal cycling pattern generated from participant-specific cadence and cycling duration. Cycling rhythmicity was assessed by evaluating the revolution-to-revolution variability in the time required to complete a revolution. Statistically significant differences (*p* < 0.001) using the Wilcoxon Rank Sum test were found between the two groups for both the metrics. Additionally, decision tree analysis revealed thresholds of smoothness <0.01 and rhythm <0.089–0.115 s for discriminating a less smooth, irregular cycling pattern characteristic of CP from typical cycling. In summary, the objective measures developed in this study indicate significantly less smoothness and rhythm of cycling in children with CP compared to children with typical development, suggestive of altered coordination and poor motor control. Such quantitative assessments of cycling motion in children with CP provide insights into neuromotor deficits that prevent them from cycling at intensities required for aerobic benefits and for participating in cycling related physical activities with their peers.

## Introduction

Cerebral Palsy (CP) is a neurodevelopmental disorder of movement and posture that results from an injury to the fetal or infant brain (1). Children with CP typically present with motor deficits such as altered muscle tone and muscle weakness, and may experience impaired sensory and cognitive impairments (2, 3). Although CP itself is a nonprogressive disorder of the brain, the impairments and functional limitations associated with CP are progressive, with many children becoming less independent with functional mobility as they enter their teenage years (4–6). Children and adolescents with CP participate in less habitual physical activity and are sedentary for more than twice the maximum recommended amount (7, 8). Unfortunately, many children with disabilities are unable to meet global physical activity recommendations due to functional impairments that limit the type of exercise activity they can participate in (6) as well as they may be limited from safely performing exercise or accessing the equipment needed to do so (9, 10). The disparity is often exacerbated by the interventions used to abate musculoskeletal and soft tissue changes that contribute to deformity, muscle tightness, and joint contractures. Selective dorsal rhizotomies, muscle/tendon lengthening procedures, serial casting, botulinum toxin injections, corrective bony procedures and the like, further compromise muscle strength by removing spasticity thereby unmasking muscle weakness, by putting muscles at unfavorable lengths for force generation, and by forced periods of prolonged immobility required by the corrective procedures (11–15). Thus, as children with CP mature, they have marked difficulties in maintaining fitness and functional ability. Hence, it is critical to develop exercise modalities that enable children with CP with limited or marginal ambulatory abilities to safely engage in physical activities.

Recumbent stationary cycling has been proposed as a safe, enjoyable, and practical exercise modality for children with CP that lack the postural control and strength necessary for upright exercises (16–18). Individuals with CP, however, are known to have impairments such as agonist-antagonist co-contraction and abnormal muscle tone (19), which may lead to irregular, halted progression of revolutions during cycling (20), thus affecting the rhythmicity and smoothness of cycling. Cycling with poor smoothness, e.g., arrested progression of revolutions and poor rhythmicity may result in inefficient cycling and reduced intensity of the exercise, and thereby, lead to reduced efficacy. Cycling with maladaptation will further lead to reinforcement of atypical movement patterns. Thus, it is critical to evaluate the motor control of cycling to train correct neuromuscular strategies for more optimal benefits from cycling. Although cycling performance has been previously evaluated in terms of muscle activation, kinematics and kinetics (19, 21) there are no studies that quantitatively describe motor control during cycling.

Smooth and rhythmic movements are a characteristic of well-developed motor control (22). While several smoothness metrics based on upper limb reaching movements, such as jerk (the time derivative of acceleration) and spectral analysis, have been proposed (23), they are affected to different degrees by measurement noise, movement duration, and periods of movement arrest. Using these metrics for detecting differences in smoothness during upper limb motion between healthy controls and individuals with stroke, cerebellar disorders and Parkinson's disease has led to mixed results (24). Such metrics are especially problematic in CP for a couple of reasons. First, taking higher order derivatives of abrupt, jerky movements that are characteristic in individuals with CP leads to outputs that are closer to the metric's ceiling values. This can result in reduced sensitivity of the measure during within- and between-participant comparisons. Second, most smoothness metrics do not quantify the temporal aspect of motion, such as regularity and variability in duration of cycling revolutions, which are important components of motor control. Thus, there is a need for robust, dimensionless, and sensitive measures for evaluating smoothness and rhythm of cycling in CP. Such metrics of cycling smoothness and rhythm may enable more effective corrective training strategies that could make cycling exercise more widely adapted by individuals with CP. With further rigorous testing on sufficient sample sizes, such metrics can have the potential to serve as tools to track changes in motor impairments in CP and the effect of treatments, such as functional electrical stimulation (FES) and biofeedback-augmented cycling, on improving motor control. The aim of this study is to characterize differences in motor control of cycling in children with CP and with typical development (TD) by developing novel metrics to quantitatively describe cycling smoothness and rhythm. We hypothesize that children with CP will demonstrate less smoothness and rhythm of cycling motion compared to those with typical development.

## Methods

Children with spastic diplegic CP were recruited through the outpatient CP clinic at Shriners Hospital for Children, Philadelphia and local referral sources. Appropriate Institutional Review Board, administrative permissions were obtained. Additionally, written informed consent from the parent/guardian of the participants and written assent from the participants were obtained. The data from children with TD was obtained from a pre-existing dataset of 10 healthy, typically developing children recruited in a hospital setting through advertisement at the hospitals, local community-based sources, siblings of previous participants, and word of mouth. None of the children with TD were patients at the hospital. All participants were screened by a physical therapist for the inclusion and exclusion criteria (Table 1).

**Table 1:** Inclusion and exclusion criteria.

**Inclusion criteria**	**Exclusion criteria**
• Ages 10–18^a^	• Lower-extremity orthopedic surgery or traumatic fracture within the past 6 months
• Diagnosis of spastic diplegic CP^b^	• Lower-extremity joint pain during cycling
• GMFCS II, III, or IV^b^	• Spinal fusion extending to the pelvis
• Adequate range of motion of the hips, knees, and ankles to allow pedaling	• Hip, knee, or ankle joint instability or dislocation • Lower-limb stress fractures in the past year
• Visuoperceptual skills and cognitive/communication skills to follow multiple step commands for attending to exercise and data collection	• Symptomatic or current diagnosis of cardiac disease as assessed by the American Heart Association guidelines for cardiac history • Current pulmonary disease or asthma and taking oral steroids or hospitalized for an acute episode in the past 6 months
• Ability to communicate pain or discomfort with testing and training procedures	• Severe spasticity in legs (score of 4 on the Modified Ashworth Scale)^b^ • Severely limited joint range of motion or irreversible muscle contractures that prevented safe positioning on the cycle^b^
	• Diagnosis of athetoid or ataxic CP^b^

a *Age range for participants with typical development was 13–19 years*.

b *Participants with cerebral palsy (CP) only*.

The system used for the CP group consisted of a commercially available recumbent sport tricycle (www.kmxkarts.co.uk) fitted with shank guide orthoses to control for excess hip adduction and abduction movement (Appendix A) (25). The bicycle crank and spindle assembly was instrumented with sensors to indicate crank position and cadence. The cycling assessment system for the children with TD consisted of a semi-recumbent, free-standing Restorative Therapies, Inc. bicycle (Baltimore, MD) attached to a therapy bench. The children in the CP group were all novice cyclers, and hence performed 20-min practice sessions twice daily for 3 days before the assessment while the children with TD performed a 10 min practice session. All children were allowed rest breaks as needed during the practice sessions. During the assessment, the children in the CP group cycled for an average of 30 ± 13 s (mean ± SD) while children with TD cycled for 15–30 s. Additionally, children with TD were asked to cycle at a target cadence of 60 rpm. However, the participants in CP group had difficulties in attaining the 60 rpm target cadence. Hence, they were all encouraged to pedal as fast as they could to get cycling as close to 60 rpm as possible. The ergometer resistance was calculated using the same formula in both CP and TD groups and was adapted from Doré et al. (26). Load (in newton-meters) = 0.49 N/kg × body weight (in kilograms) × crank arm length (in meters). The CP (R01HD062588) and TD datasets (19) were from two separate larger studies. Despite the different systems for children with CP and TD, the overall set-up was custom adjusted according to the same specifications for each participant based on their anthropometric data (Appendix A). Because the same standardized system set-up, including crank arm length, seat-to-pedal distance and seat-to-greater trochanter distance, were used for both the groups, we do not expect the different cycling systems to contribute appreciably to the between-group differences that may be observed. Data were analyzed using customized software (MatLab, The Mathworks, Inc.) and statistical software (JMP^®^, Version 14.3.0, SAS Institute Inc.).

### Data Analysis

**Figure 1:**
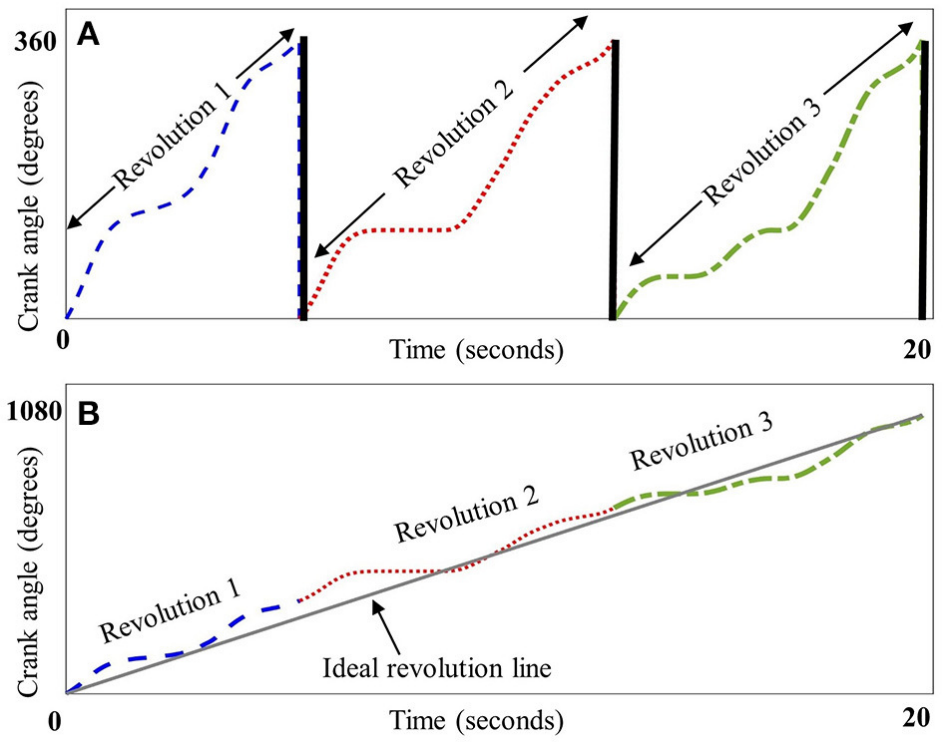
Schematic representation of crank angle smoothness. Panel **(A)** depicts crank angle (plotted against time in seconds) for three representative revolutions from a child with CP, each dashed section depicting one revolution from 0° to 360°, thick black lines indicate the discontinuity between 360° and 0° at the end of each revolution. Panel **(B)** depicts the concatenation of these revolutions, resulting in a linear form that was cross-correlated with a line depicting an ideal, smooth revolution (straight gray line).

Crank angle data were lowpass filtered at 5 Hz and plotted against time, the result being a sawtooth waveform indicating the angle of the recumbent cycle's crank as the trial progressed. To eliminate potential pedal acceleration and deceleration influences, the first and the last revolution of the crank were discarded. As crank angle data are circular, there is a discontinuity every time the angle value crosses from 360°→0° (Figure 1A). To eliminate this discontinuity, crank angle was converted from repeating 0–360° epochs to a linear form by concatenating the angle data and appending them in series. The resultant angle-in-series data was a time series representing the angular progression of crank from zero to 360 × the number of revolutions (Figure 1B). To quantify the deviation of each participant's angle-in-series from the smoothest possible crank angle, the angle-in-series was cross-correlated with a straight line that connected the beginning to the end of angle-in-series' data points. This straight line, considered the participant -specific ideal crank angle, represented the smoothest transition from 0°→360°. The duration of this ideal straight line for each cycling trial was the same as the cycling duration of the observed pattern to account for possible influences of the cycling speed and duration on smoothness. Also, to eliminate any influence of cadence on smoothness, the number of revolutions in the ideal pattern were the same as that in the observed cycling trial. Thus, an ideal cycling pattern was “custom-made” for each participant based on their own speed, cycling duration and cadence.The calculation of the cross-correlation between angle-in-series and ideal crank angle for the time lag *n*, including the formula used to calculate it, is further described in Appendix B. The maximum of the cross-correlation of the angle-in-series and ideal crank angle was then normalized to the maximum of ideal line's autocorrelation, which is the cross-correlation of the signal with itself, to make it dimensionless for better comparison. The results were expressed as the smoothness measure. Higher values indicate less smooth cycling motion.

To quantify the temporal characteristics of cycling, or in other words, to assess how rhythmic and regular the cycling pattern was, the variability of the time taken for completing each revolution in a cycling trial was measured by computing its standard deviation. Therefore, similar to the definition of gait rhythmicity as stride-to-stride variability in gait timing (27, 28), we defined cycling rhythmicity as revolution-to-revolution variability in the time required to complete a revolution. Thus, the higher the standard deviation, the higher the variability and lower the rhythmicity of each revolution.

We analyzed between-group differences by performing a nonparametric Wilcoxon Rank Sum test. To further support the ability of smoothness and rhythm metrics to discriminate between the typical cycling pattern and a less smooth, irregular pattern seen in CP, we performed a decision tree analysis using the Partition routine within JMP using default settings. The ability of a decision tree to accurately classify group membership is enhanced by individual measures with distributions immediately distinguishable between groups and can be refined further with additional measures that explain group membership conditional on earlier branches in the tree. Decision trees were built separately, using smoothness or rhythm for the initial branch split, to determine threshold values to distinguish between CP and TD cycling patterns, and then refined if possible by the remaining predictor. In cross validation, validation sets were randomly formed with ~80% of the data used for training the algorithm and establishing the decision rules and the remaining ~20% used as a validation set on which the rules could be applied. We replicated the process three times to probe the sensitivity of the fit to the random allocation of training and validation. An additional probe of sensitivity was conducted using the JMP software implementation of 5-fold cross validation, which we also ran three times for each predictor to build confidence in the approach through the generalized *R*^2^ reported. Confusion matrices report the number of correct and incorrect predictions for CP/TD cycling pattern using decision tree-derived thresholds for smoothness and rhythm metrics.

Lastly, to explore the sensitivity of our metrics to aberrant revolutions, we performed simulation analysis using custom MATLAB software. We generated alternate datasets from the original dataset in the following way:

1. To explore how a single aberrant revolution affects smoothness, we removed the most aberrant cycle in terms of smoothness, i.e., the most unsmooth revolution from each participant's trial. Thus, we generated an alternate dataset from the original dataset without the most unsmooth revolution.
2. To explore how a single aberrant revolution affects rhythm, we repeated the same process for rhythm, where we generated an alternate dataset without the revolution with worst rhythmicity for each participant.
3. To investigate how the order in which the aberrant revolution occurred in a trial affects smoothness, we generated an alternate dataset by shuffling the positions of the revolutions in a trial.

Next, we recalculated the smoothness and rhythm values for the alternate datasets mentioned above. The difference between the two datasets was analyzed using paired *t*-tests, where the original and alternate values for each participant formed a single pair. Because shuffling the revolutions would not change the variability of the revolutions and in turn would not change the rhythm values, no further statistical analysis was performed for rhythm for the third scenario listed above.

## Results

**Figure 2:**
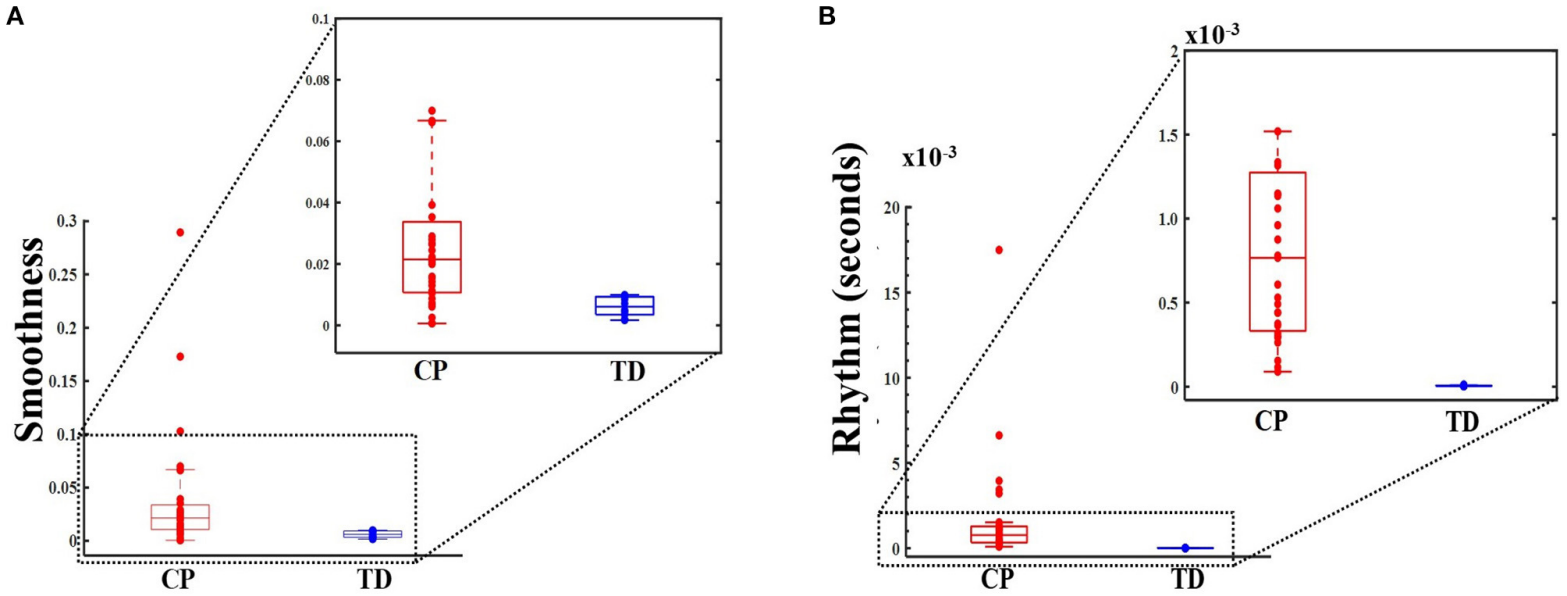
Box and whisker plots, with scattered values (dots) indicating each data point, show increased values, i.e., less smoothness (dimensionless metric) in panel **(A)** (top panel) and for rhythm (in seconds) in panel **(B)** (bottom panel) for CP group (on the left, in red) compared to typically developing (TD) group (on the right, in blue). Higher smoothness values imply a less smooth cycling pattern. Higher rhythm values imply increased variance, and hence, poorer rhythm. The boxes depict the 25th−75th quartile and the horizontal line depicts the median. Inset: Smoothness and rhythm values for majority of the data (excluding the extreme values).

Thirty-one ambulatory adolescents with CP were recruited, with Gross Motor Function Classification System (GMFCS) levels II–IV (level II, III, and IV had 10, 10, and 11 participants, respectively). There were six females in the CP group and seven females in the TD group. There were no significant between-group differences for age (*p* = 0.127) and BMI (*p* = 0.570). The mean [standard deviation (SD)] age was 13.7 (2.6) years for children with CP and 14.9 (1.4) years for children with TD. The mean (SD) BMI was 20.3 (5.5) kg/m^2^ for children with CP and 22.6 (5.4) kg/m^2^ for children with TD. By inspection, Figure 2 boxplots reveal that the distributions for smoothness and rhythm each appear different for children with CP and TD (Figure 2). Extreme observations or outliers were cross-checked through visual inspection of the raw data and visualization of the crank angle against time, which revealed that these were valid measurements and not measurement errors. The two-sided tests yielded normal approximation *z*-values of −3.81 (Smoothness) and −4.69 (Rhythm), each statistically significant (*p* < 0.001). The mean smoothness and rhythm (mean ± standard error) for children with CP [0.039 ± 0.010 (dimensionless) and 1.672 ± 0.583 (s), respectively] were significantly higher than that for children with TD (0.006 ± 0.001 (dimensionless) and 0.005 ± 0.001 (s) respectively). Higher values for both metrics indicate less smoothness and less rhythmicity of cycling motion.

We explored the potential of our measures to accurately discriminate the cycling pattern as being that of a child with CP or TD. Once either smoothness or rhythm was included in the decision tree analysis model, the second metric added no additional predictive advantage, resulting in a single decision rule for each metric. The decision rule for smoothness revealed smoothness >0.01 as threshold for predicting cycling pattern characteristic of the CP group for all validation sets. The decision rule for rhythm revealed rhythm >0.115 s as threshold for predicting a CP cycling pattern for validation set 1 and >0.089 s for validation sets 2 and 3. Additionally, a software generated five-fold cross validation on the same data yielded a generalized *R*^2^ = 0.99 in each of the three runs. The details about the training and validation confusion matrices for the decision tree are depicted in Appendix C.

Our exploration of the sensitivity of the metrics showed that there were no significant differences between the original smoothness values and the values generated after removing the most unsmooth revolution (*t* = −0.287, df [40], and *p* = 0.776). There were, however, significant differences between the original rhythm values and the values generated after removing the revolution with the worst rhythmicity (*t* = 2.594, df [40], and *p* = 0.013). On repeating the same analysis after excluding the participants whose trial had <12 revolutions, there were no significant differences between the original rhythm and the rhythm without the most aberrant cycle (*t* = 1.580, df [19], and *p* = 0.065). Lastly, the shuffling of revolutions did not yield smoothness values that are statistically significant from each (*t* = 1.072, df [40], and *p* = 0.145).

## Discussion

The purpose of this study was to develop objective measures to quantify motor control during cycling in children with CP and with TD. We developed two measures, one to assess the quality of cycling motion i.e., smoothness, and the second to assess the regularity in the timing of cycling, i.e., rhythm of cycling motion.

### Differences Between CP and TD Cycling

**Figure 3:**
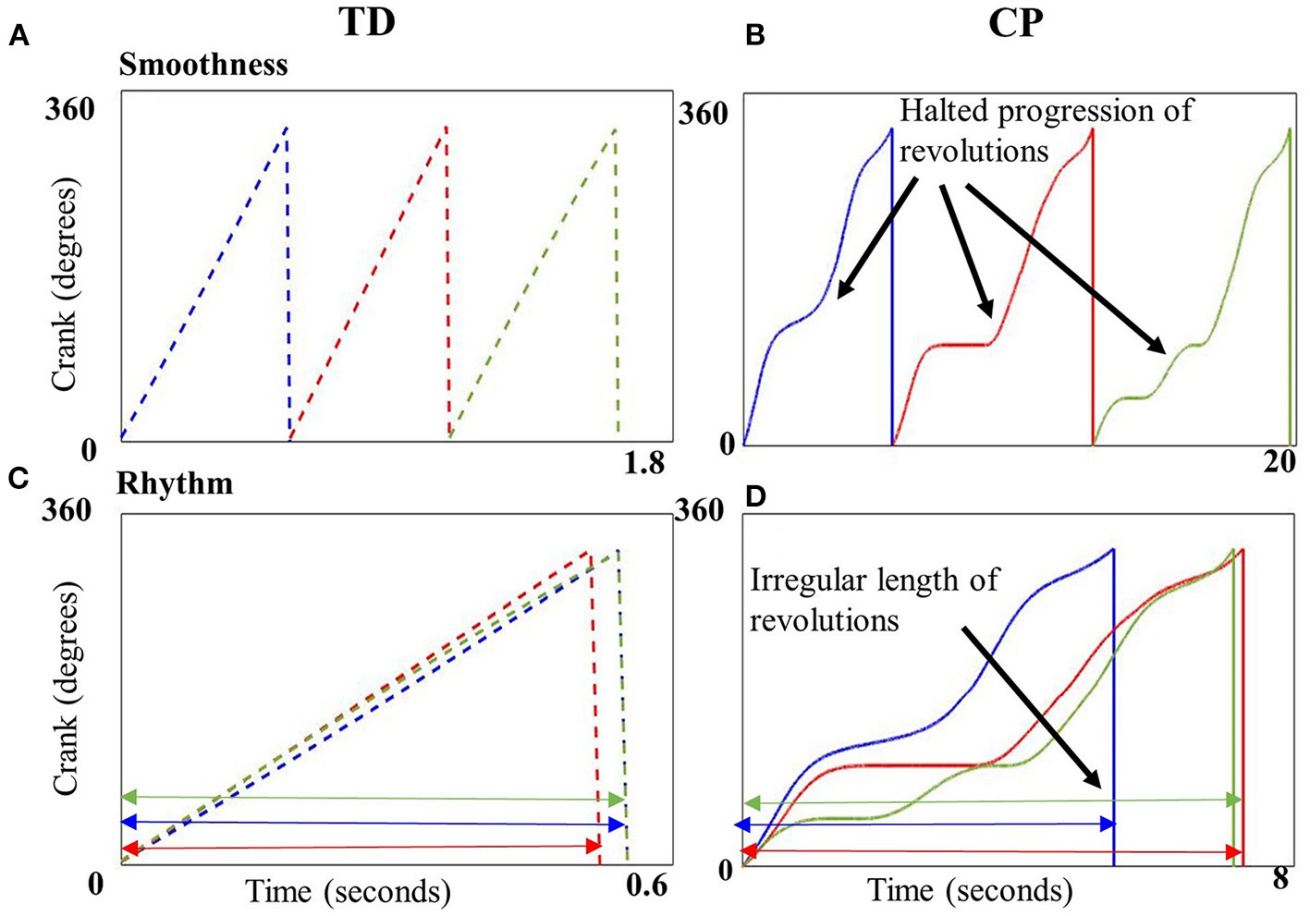
Crank angle plotted against time (seconds) for three example revolutions (blue, red, and green) from a single representative child with typical development (TD) **(A,C)** and CP **(B,D)**. Top panels depict the smooth transition from 0 to 360° in a child with TD **(A)** vs. the uneven, halted progression in a child with CP **(B)**. Cycling revolutions are superimposed on each other in the bottom panels to depict rhythmicity, which shows the consistent length of the revolutions in a child with TD **(C)**, implying better rhythmicity in contrast to the inconsistent length of revolutions in a child with CP **(D)** implying poor rhythmicity.

Our results show that children with CP cycled with significantly less smoothness as compared to children with TD (Figures 3A,B). Thus, the progression of crank angle from 0° to 360° was significantly more halted and abrupt in children with CP. Also, children with CP cycled with significantly less rhythmicity compared to children with TD, i.e., the time taken to complete a cycling revolution was extremely variable in the CP group, leading to irregularity and poor rhythmicity of the motion (Figures 3C,D). Thus, both metrics were able to quantify the difference in motor control of cycling between children with CP and TD. These differences may be due to agonist-antagonist co-contraction, increased duration of muscle activation and altered motor strategies previously reported in children with CP during cycling (19, 21). Our results are also consistent with reports of reduced smoothness during upper limb reaching in CP (29) and with video analysis that showed irregular time periods spent within different quadrants of the pedaling cycle (20). Our results collectively with these studies are indicative of altered motor control in CP.

Additionally, the decision tree results further support the ability of the two outcome measures to successfully discriminate between a typical cycling pattern and an abnormal, less smooth, and arrhythmic cycling pattern seen in CP. The decision tree analysis identified empirically derived thresholds for these measures. Smoothness above 0.01 was attributed to the CP group while smoothness below 0.01 was attributed to the typical cycling pattern. Similarly, rhythm scores above 0.089 and 0.115 s distinguished a CP cycling pattern from TD. Obtaining two threshold values from two different training sets for rhythm is not unusual, given the small data set with high variability in the CP group which comprised individuals with different functional capabilities (GMFCS levels II–IV). However, the results of the rhythm confusion matrices are encouraging (Appendix C).

### Sensitivity of the Metrics

Exploration of the sensitivity of the metrics revealed that the smoothness values were largely unaffected by a single aberrant cycle, implying that while the metric can consistently discriminate between a smooth and unsmooth cycling pattern, it is less likely to be influenced by a single aberrant revolution or an outlier. The rhythm metric significantly changed due to the removal of the most aberrant cycle, implying that it is extremely sensitive to even a single aberrant revolution. However, when the participants with <12 revolutions were excluded from the analysis, a single aberrant revolution was less likely to affect its value. Thus, rhythm is especially more sensitive to deviations caused by single outlier in the absence of sufficient number of cycling revolutions. We caution against using twelve revolutions as an absolute threshold or rule of thumb for collecting the minimum number of revolutions, rather our intent was to demonstrate that too few cycling revolutions might magnify the effect of single aberration on the metric. While another approach to characterizing variability, such as using the coefficient of variation, which is standard deviation divided by mean, may be used to quantify rhythm, it may mask the raw variability that the standard deviation captures. As both standard deviation and mean may simultaneously increase or decrease, the resultant coefficient of variation may remain the same, masking potential pre- to post-intervention changes for a patient. Finally, shuffling of the revolutions in a trial did not affect either metric, implying that the metrics are not affected by the location of the aberrant revolution.

Existing smoothness metrics, which are especially sensitive to signal-to-noise ratios, result in different smoothness values for the same movement pattern with changes in movement speed. This is because slower movements have lower SNR (signal to noise ratio) than faster movements. Thus, if the smoothness measure is extremely sensitive to changes in SNR, then one would get different results for smoothness of the same cycling pattern at different speeds. The strength of our smoothness metric lies in comparing the observed cycling motion with a “custom-made” participant-specific ideal cycling pattern derived from their own cycling speed, duration, and cadence, thus making possible comparisons across individuals with different instantaneous speeds and cadences. This attribute is especially important while assessing motion in a clinically heterogeneous disorder such as CP, where individual may vary vastly in their functional abilities, leading to different cycling speeds and durations. Our smoothness metric, in essence, enables the evaluation of motor control of the cycling motion, irrespective of the cycling speed and cadence.

### Clinical Application for Enhancing Physical Activity

Quantitative assessment of motor control during cycling may provide insights into some of the potential impairments, such as poor rhythmicity and halted unsmooth motion that may hinder a child from cycling at higher intensities. Development of outcome measures like the smoothness and rhythm metrics is the first step toward quantitative assessment of motor control.

Both metrics are computationally inexpensive, clinically intuitive, and can be used to assess abrupt, jerky movements. More importantly, these metrics give us a snapshot of the cycling “quality” (e.g., irregular, halted, abrupt motion) over metrics that only measure cycling “quantity” (e.g., duration of cycling, cycling speed etc.). Thus, a child cycling with a smoother, more rhythmic motion after undergoing a rehabilitation program may demonstrate improved motor control rather than a child who may be cycling faster or for longer duration albeit with compensatory, maladaptive motions (e.g., backpedaling, arrested motion). If metrics to quantify the quality of motion are unavailable, then these compensatory motions may go unchecked and be reinforced over the training duration. The metrics in this study may help in identifying and targeting these deficits. For example, poor smoothness scores during cycling may indicate a need to address muscle spasticity and co-contraction in order to improve their cycling motion while poor rhythmicity may indicate a need to use metronomes or auditory cues at portions of the cycling revolution to ensure regular, rhythmic motion. Thus, these metrics may aid in designing rehabilitation programs to meet physical activity needs of not just children with cerebral palsy but other neurodevelopmental disorders as well. Additionally, the smoothness and rhythm thresholds derived from a decision tree analysis, potentially supported by a larger study, might serve as post rehabilitation targets for a cycling program for children with CP.

Due to impairments such as altered muscle activations patterns, agonist–antagonist co-contraction, and abnormal timing of activation during cycling, children with CP demonstrate an irregular, halted cycling pattern (19–21). Thus, they may be less likely to generate smooth and symmetric motion required to attain a high cycling intensities needed to attain cardio-respiratory benefits. The World Health Organization's International Classification of Function, Health, and Disability (ICF) model stresses the importance of incorporating a child's social and environmental needs into rehabilitation programs. Hence, it is critical to implement rehabilitation programs that incorporate functional activities that a child is personally motivated to perform and that improve their participation in family and social activities. Cycling provides a great way of addressing body structure and function components of ICF as well as encouraging participation in an activity that can be performed outside of the PT clinic using an adapted cycle with family and friends. The smoothness and rhythm metrics in this study provide an avenue to clinicians to quantitatively assess an “activity” rather than the traditional outcome measures that may be subjective or may evaluate a single plane movement. Improved ability to cycle smoothly and rhythmically may encourage participation of children with CP with their typically developing peers, siblings and friends in a socially enjoyable physical activity. Children with CP are more likely to participate in a physical activity if it lets them “fit in” and may be discouraged if the motor tasks are too challenging or make their disability or asymmetries in motion stand out (9, 30). Additionally, parents perceive symmetrical movements during physical activity as critical (30). By enabling smoother, rhythmic and in turn symmetric cycling motion, children may be more motivated to participate in a physical activity with higher confidence and self-esteem. Not only will this help in addressing the social development of children with CP but they can engage in an enjoyable activity that is not viewed as “exercise.”

Lastly, it is important to note the “chicken and egg” problem of higher physical activity and smoother motion i.e. children with irregular and asymmetric motion are less likely to participate in physical activities whereas children with better motor abilities may find it easier to engage in physical activities (30, 31). Conversely, children with higher physical activity levels show better motor performance and motor learning abilities (32, 33) and hence, may have smoother, more rhythmic movements. We attempt to take the first step toward addressing this problem by developing metrics to analyze and with further development, correct such maladaptive motor behavior during cycling.

### Limitations

There are some limitations to consider when interpreting the results of this study. Firstly, the data for each group were collected as a part of two separate studies and this may have introduced potential between group differences. While there were no significant differences between the ages for the two groups, overall the participants in the CP group were slightly younger than those in the TD group. The small difference in age combined with developmental changes occurring during the early teens and the onset of puberty may contribute to potential inter-group differences. Also, children with CP were asked to achieve a target cadence of 60 rpm while children with TD were asked to pedal as fast as they could. While the smoothness metric is unaffected by inter-participant differences in cycling cadences, the differences in cycling rhythm may be magnified or reduced. At this point, we do not know definitively the implications of the different cadences on the cycling rhythmicity and acknowledge it as a potential factor to consider when interpreting our results.

Secondly, an important limitation to consider is that because the study only looked at the differences in children with and without CP, which one might expect are more obvious, we do not know yet if these metrics can detect extremely small, subtle changes in smoothness and rhythm. Children with CP being novice cyclers might show starker differences when compared to children with TD, which may have had some previous experience of cycling. While we gave the CP group more practice sessions than TD to account for potential previous cycling experiences in participants in the TD group, the novelty of the cycling task for children with CP may still contribute to the lack of smoothness and rhythm seen in this group.

While the sample size of our study was relatively small, these results show that our smoothness and rhythm measures hold promise as novel outcome measures deserving of further study to quantify motor control during cycling in children with CP. The decision tree models explored here show potential for being able to classify CP vs. TD based on smoothness or rhythm. However, with so few samples, the threshold for separation that is derived from a nonparametric split along an axis is inherently coarse and variable. To gain confidence in a fitted threshold from this process, a much larger study is needed where we would expect greater density of observations in the region where a best split would occur and therefore a finer, less variable fitted threshold for classification. Future work with larger sample sizes and stratified sampling for GMFCS levels will be needed to establish the sensitivity and discriminatory ability of these metrics on a sample with different cycling and functional abilities. Additionally, future studies that establish testing criteria such as minimum required number of cycling revolutions in a trial, the effect of different cycling cadences particularly on rhythm will be beneficial to standardize the testing process for clinical use.

In summary, this study identified two novel objective measures for quantifying cycling performance by assessing smoothness and rhythm of cycling. These measures may indicate neuromotor differences during cycling in children with CP compared to their TD peers. In particular, significantly less smoothness and rhythm of cycling in children with CP as compared to TD might indicate poor timing and irregularity of movement, altered coordination and motor control. These measures are offered as potential markers for tracking progression of motor control deficits and maybe used to evaluate effects of intervention during cycling training in children with CP.

## Data Availability Statement

The raw data supporting the conclusions of this article will be made available by the authors, without undue reservation.

## Ethics Statement

The studies involving human participants were reviewed and approved by the Institutional Review Boards at Temple University and University of Delaware. The patients/participants provided their written informed consent to participate in this study. Written informed consent to participate in this study was provided by the participants' legal guardian/next of kin.

## Author Contributions

AS and AB: conception and design of the work. AB, TJ, and SL: data collection and critical revision. AS, AB, and BB: analysis of data and interpretation. AS: drafting the work. AS, AB, TJ, BB, and SL: final approval of the work. All authors agree to be accountable for the content of the work.

## Funding

This study was supported by NIH (R01HD062588), Shriners Hospitals for Children (Grant #8530), and from a Clinical Research Grant from the Pediatric Section of the American Physical Therapy Association.

## Conflict of Interest

The authors declare that the research was conducted in the absence of any commercial or financial relationships that could be construed as a potential conflict of interest.

## Publisher's Note

All claims expressed in this article are solely those of the authors and do not necessarily represent those of their affiliated organizations, or those of the publisher, the editors and the reviewers. Any product that may be evaluated in this article, or claim that may be made by its manufacturer, is not guaranteed or endorsed by the publisher.
