# Supplementary material for: Characterizing Cycling Smoothness and Rhythm in Children With and Without Cerebral Palsy
Source: Front Rehabil Sci. 2021 Sep 7;2:690046. doi: 10.3389/fresc.2021.690046 (PMC9397803; doi:10.3389/fresc.2021.690046)
Supplement: Supplementary file 1 [file Data_Sheet_1.DOCX]

**APPENDIX A (SUPPLEMENTARY MATERIAL)**

**Figure A**: Panel **A** depicts the bicycle set-up for CP group using an instrumented commercially available recumbent sport tricycle ([www.kmxkarts.co.uk](http://www.kmxkarts.co.uk)) fitted with a shank guide. The TD group used a comparable set-up using a Restorative Therapies, Inc. (Baltimore, MD) free-standing bicycle attached to a therapy bench as depicted in panel **B**. All components in both set-ups were adjusted based on subject anthropometrics (panel **C**). (1) Seat-to-pedal distance = 85% of the distance from the greater trochanter to the base of the calcaneus. (2) Seat-to-greater trochanter distance = 15% of the distance from the greater trochanter to the base of the calcaneus. (3) Crank arm length = 30% of tibial length. Panel **B** is reproduced from Johnston, Prosser, & Lee. Differences in pedal forces during recumbent cycling in adolescents with and without cerebral palsy. Clin Biomech (Bristol, Avon). 2008 Feb; 23(2): 248–251. by permission of Elsevier. Panel **C** is reproduced from Johnston, Barr, & Lee. Biomechanics of Submaximal Recumbent Cycling in Adolescents With and Without Cerebral Palsy, Physical Therapy, (2007) 87(5), 572-585 by permission of Oxford University Press.
