# Supplementary material for: Characterizing Cycling Smoothness and Rhythm in Children With and Without Cerebral Palsy
Source: Front Rehabil Sci. 2021 Sep 7;2:690046. doi: 10.3389/fresc.2021.690046 (PMC9397803; doi:10.3389/fresc.2021.690046)
Supplement: Supplementary file 2 [file Data_Sheet_2.DOCX]

**APPENDIX B (SUPPLEMENTARY MATERIAL)**

Cross-correlation measures the correlation between two signals as one of them slides through the other with varying time lags (Fig. B). Thus, cross-correlation was used as a measure of similarity between two signals as a function of the time-lag between them, in applications such as medical image processing (1, 2). The peak of cross-correlation occurs at the time lag that the two signals are correlated the most. Thus, in the biomedical applications the technique was mostly used to measure the phase difference, i.e., the time lag between two signals (3). The amplitude of the peak depends on the peak and length of the signals; however, if dimensionless, it can be used to show the level of similarity between the two signals. Thus, we proposed a smoothness measure based on a dimensionless cross-correlation of the ideal crank angle and the angle-in-series signal. Note that the ideal crank angle line is different from the best linear fit to the angle-in-series as this line connects the beginning to the end of the crank-in-series.

In 1D signals, i.e., time series, cross-correlation is the sliding dot product of the two signals (Eq. 1). Therefore, for our time series, *AiS(m)* and *Idl(m)*, the angle-in-series and the ideal crank angle respectively, cross-correlation for the time-lag *n* is defined as

1. $corr\left( AiS,Idl,n \right)=\sum_{m=-N}^{+N} AiS\left( n+m \right)Idl(m)$

Note that the length of both signals is N (4x10^4^ in Fig. A). The ideal crank angle is stationary, and the angle-in-series dislocates in time over it, producing a signal of length 2N where each sample is the summation of the sample-by-sample multiplication of the overlapping area (Fig. A). The peak occurs when the two signals overlap optimally. Thus, the peak is at no time lag, *n*=0 (Fig. A.3), for our smoothness measure, where the positive peaks of signals *AiS(m)* and *Idl(m)* are aligned, and thus maximally contributing to *corr*. In the ideal scenario, where the *AiS(m)* and *Idl(m)* the peak (Eq. 2) is at *n*=0.

1. ${Peak}_{Idl}=\sum_{m=-N}^{+N} {AiS}^{2}\left( m \right)$

To make the smoothness measure dimensionless for a more effective comparison, we normalized the peak of the *corr* to the peak of the ideal scenario (Eq. 3). Note that because the *Peak_Idl_* (Eq. 2) and the *corr* (Eq. 1) has the same dimension Eq. 3 has a dimensionless value. In an ideal scenario maximum value of Eq. 1 is equal to Eq. 2 and Eq. 3 is equal to one. Therefore, the absolute value of the (Eq. 3) was subtracted from one, at the end, to calculate its distance from the ideal scenario. Eq. 4 shows our proposed formula to calculate smoothness value based on cross-correlation.

Figure B. In time series, cross-correlation is the sliding dot product of the two signals. In the smoothness measure the ideal crank angle (the black triangle) is stationary, and the angle-in-series (the colored triangle) dislocates in time over it. At each step cross-correlation is the summation of the sample-by-sample multiplication of the overlapping area. Not that, for both signals, length, N is 4x10^4^. (1), (2), (3), and (4) depict the time lag, n, of -N, -N/2, 0, and N/2, respectively. (3), the cross-correlation’s peak occurs when the two signals overlap optimally, n=0.

1. $\frac{max\{corr\left( AiS,Idl,n \right)\}}{{Peak}_{Idl}}$
2. $smoothness=\left| 1-\frac{max\{corr\left( AiS,Idl,n \right)\}}{{Peak}_{Idl}} \right|$

References:

1. Quiroga, R. Quian, et al. "Performance of different synchronization measures in real data: a case study on electroencephalographic signals." *Physical Review E* 65.4 (2002): 041903.

2. Penney, Graeme P., et al. "A comparison of similarity measures for use in 2-D-3-D medical image registration." *IEEE transactions on medical imaging* 17.4 (1998): 586-595.

3. Potas, Jason Robert, et al. "Waveform similarity analysis: A simple template comparing approach for detecting and quantifying noisy evoked compound action potentials." *PloS one* 10.9 (2015).
