# Supplementary material for: Characterizing Cycling Smoothness and Rhythm in Children With and Without Cerebral Palsy
Source: Front Rehabil Sci. 2021 Sep 7;2:690046. doi: 10.3389/fresc.2021.690046 (PMC9397803; doi:10.3389/fresc.2021.690046)
Supplement: Supplementary file 3 [file Data_Sheet_3.DOCX]

**APPENDIX C (SUPPLEMENTARY MATERIAL)**

Smoothness

Rhythm

**Appendix C.** Training and Validation Confusion Matrices for Validation Sets 1-3 (top to bottom) for Smoothness (left) and Rhythm (right) show that for rhythm, only one misclassification was reported over the three validation sets and none for training. For smoothness, 1-2 misclassifications were reported across validation and training sets. Thus, these matrices depict the ability of both metrics as classifiers to distinguish between TD and CP cycling.
